# The length of hospital stays and clinical and therapeutic characteristics of patients with COVID-19 early in the pandemic in Taif City, KSA: A retrospective study

**DOI:** 10.1097/MD.0000000000032386

**Published:** 2022-12-23

**Authors:** Maram Abduljabbar, Raghad Alghamdi, Kholoud Althobaiti, Shumukh Althubaiti, Najla Alharthi, Ghada Alosaimi, Mawddah Qunq, Lobna Saleh, Manal Alosaimi

**Affiliations:** a Department of Pharmacology and Toxicology, Collage of Pharmacy, Taif University, Taif, Saudi Arabia; b Pharm D, College of Pharmacy, Taif University, Taif, Saudi Arabia; c Department of Pharmacology and Toxicology, College of Pharmacy, Taif University, Taif, Saudi Arabia; Addiction and Neuroscience Research Unit, College of Pharmacy, Taif University, Taif, Saudi Arabia; Department of Clinical Pharmacology, Faculty of Medicine, Ain Shams University, Cairo, Egypt; d Department of Basic Science, College of Medicine, Princess Nourah Bint Abdulrahman University, Riyadh, Saudi Arabia.

**Keywords:** antibiotic therapy, antimalarial, antiviral, COVID-19, length of hospital stay

## Abstract

The coronavirus disease-2019 (COVID-19) pandemic is unprecedented in the healthcare sector worldwide. This retrospective study focused on the length of hospital stay and clinical and therapeutic characteristics of patients with COVID-19. Retrospective data of severe acute respiratory syndrome coronavirus 2 (SARS-COV-2) positive patients were collected between March 12 and June 30, 2020, and categorized into mild, moderate, and severe disease groups based on symptoms and severity of COVID-19. A total of 843 SARS-COV-2-positive patients were identified in this study (mildly symptomatic, 132; moderately symptomatic, 168; severely symptomatic, 17). The mean lengths (days) of hospital stay of Groups 1 to 8 were 16.38, 13.18, 13.72, 9.30, 6.96, 10.86, 5.77, and 7.37, respectively. Treatment Group 1 had the highest mean. In the treatment group, 7 patients who were not treated had the shortest stay. The patients with heart failure and Group 1 received antiviral, antimalarial, and antibiotic therapy; patients in Group 3 received antimalarial and antibiotic therapy; patients in Group 4 received antiviral and antibiotic therapy were tended to have a longer hospital stay. The length of hospital stay and clinical and therapeutic characteristics are crucial indicators of pandemic management, a shorter hospital stay is a positive outcome of better COVID-19 management.

## 1. Introduction

Coronaviruses belong to the Coronaviridae family of the order Nidovirales. The name “coronavirus” originates from the corona or crown-like spikes on the outer surface of the virus.^[1]^ Coronaviruses are accurate in size (65–125 nm in diameter) and contain single-stranded ribonucleic acid (RNA) as a nucleic material ranging from 26 to 32 kilobases in length, which is the largest known RNA virus genome.^[1]^ The subgroups of the coronavirus family are alpha (α), beta (β), gamma (γ), and delta (δ) coronavirus.^[1]^ The incubation period of a coronavirus varies but generally lasts up to 2 weeks.^[2]^ Although coronaviruses are ubiquitous, SARS-CoV-2 was first detected in December 2019 in Wuhan, a city in Hubei province, China, with a population of 11 million, after an outbreak of pneumonia without obvious cause.^[3,4]^

The virus, which has now spread to more than 200 countries and territories worldwide, was characterized by the World Health Organization as a pandemic on March 11, 2020.^[3,4]^ On January 10, 2021, there were 88,828,387 laboratory-confirmed cases of coronavirus disease-2019 (COVID-19) infection globally, with 1926,625 reported deaths. The number of cases and deaths outside China exceeded those within the country on March 16, 2020.^[5]^ According to the World Health Organization epidemiological update on September 28, 2022, the number of weekly new cases of COVID-19 decreased by 11% compared to the previous week, with more than 3 million new cases reported.^[6]^ Generally, the number of cases and deaths has decreased or remained stable. However, updated data on cases and deaths should be carefully interpreted because certain countries alter the policy on testing strategies, resulting in a lower overall number of tests performed and thus, a lower number of cases detected.^[6]^

COVID-19 can cause symptoms such as fever, dry cough, dyspnea, weakness, and lymphopenia, and in extreme cases, may lead to severe acute respiratory syndrome (SARS) and even death.^[7–9]^ Most people infected with SARS-CoV-2 recover quickly in a few weeks. However, health problems, such as fatigue, shortness of breath, coughing, joint pain, and chest pain, may occur in some people.^[10]^ Some may continue to suffer for a long time – a situation called “post-COVID-19 syndrome” or “long-term COVID-19.” This is especially true for the elderly and people with chronic diseases.^[10]^

After 6 months of acute infection, survivors of COVID-19 suffered mainly from fatigue or muscle weakness, sleep difficulties, anxiety or depression, fast heartbeat, and problems with memory and concentration. Moreover, in patients with critical cases of COVID-19, blood clots form faster, leading to the formation of small clots that block blood vessels that may also occur in the kidneys, which some experts call “small clots in the kidney tissue.” Furthermore, there is scientific evidence that the virus can attack the kidneys directly, and if a patient with COVID-19 develops pneumonia and needs respiratory support, a large amount of water accumulates around the lungs. In this case, doctors administer diuretics to patients to help draw fluid out of the body.^[10]^

To date, studies have shown that the origin of the virus is associated with the food market in China (Wuhan city); however, specific animal associations have not been confirmed.^[11]^ Genomic analysis discovered that SARS-CoV-2 is phylogenetically associated with SARS-like bat viruses. It is transmitted rapidly from person to person on a large scale.^[12]^

The main complications reported in patients with SARS-CoV-2 infection include cardiovascular complications, such as acute heart failure, left ventricular dysfunction, myocardial injury, myocarditis, arrhythmias, and heart failure,^[13]^ as well as neurological complications, such as anosmia, ageusia, impaired consciousness, seizures, encephalopathy,^[14]^ and stroke.^[15]^

In addition to the availability of several effective COVID-19 vaccines to reduce the incidence of COVID-19 complications, such as hospitalization and death, antiviral agents have been approved. On October 22, 2020, the FDA approved the first antiviral agent, remdesivir, for patients with COVID-19 who needed hospitalization. On December 22, 2021, the FDA issued an Emergency Use Authorization for the emergency use of paxlovid tablets (nirmatrelvir and ritonavir) for mild-to-moderate COVID-19 in cases of positive results of direct SARS-CoV-2 testing or who are at high risk of hospitalization and death due to COVID-19.^[16]^ Some therapeutic medications are used off-label, including antiviral medications,^[17]^ chloroquine/hydroxychloroquine,^[18]^ azithromycin,^[19]^ corticosteroids,^[20]^ and immunoglobulins.^[21]^

To address the gaps in knowledge mentioned above and given the ongoing spread of COVID-19 in the Middle East, this study aimed to describe the therapeutic characteristics of COVID-19 in a selected cohort of patients in Taif, Saudi Arabia, at the beginning of the COVID-19 pandemic and to identify the length of hospital stay among patients with COVID-19.

## 2. Materials and Methods

### 2.1. Study design and participants

This was a retrospective study of patients diagnosed with COVID-19 in 2 center case series, King Fasil Hospital and King Abdulaziz Specialist Hospital. We extracted data from patients admitted to King Fasil Hospital and King Abdulaziz Specialist Hospital who were confirmed SARS-COV-2 positive with polymerase chain reaction between March 12, 2020, and June 30, 2020.

### 2.2. Data collection

Data were extracted from paper and electronic records, using a unique medical record number for each patient. The data extracted included patients’ demographics, comorbidities, history of recent travel, and history of contact with a confirmed case of COVID-19 in the previous 2 weeks. In addition, the disease characteristics of COVID-19, symptoms, and severity were collected, along with the pharmacological treatment received.

### 2.3. Ethics approval

This study was approved by the Institutional Review Board, Department of Health Affairs, Taif, Ministry of Health (No. 544). Written informed consent was waived by the ethics committee.

### 2.4. Study variables

#### 2.4.1. Patient characteristics.

We classified patients according to the number of symptoms and severity of COVID-19, such that: patients with mild disease were defined as having 3 to 5 symptoms (fever, sore throat, headache, fatigue, myalgia, diarrhea, vomiting, loss of smell, runny nose, dry cough); patients with the moderate disease were defined as having more than 5 symptoms (fever, sore throat, headache, fatigue, myalgia, diarrhea, vomiting, loss of smell, runny nose, dry cough); and severe disease was defined as any patient admitted to the intensive care unit who had mechanical ventilation or oxygen inhalation.

#### 2.4.2. Patients and treatments.

We classified the patients into 8 groups according to treatment. Patients in Group 1 received antiviral, antimalarial, and antibiotic therapy; patients in Group 2 received antiviral and antimalarial therapy; patients in Group 3 received antimalarial and antibiotic therapy; patients in Group 4 received antiviral and antibiotic therapy; patients in Group 5 received antiviral therapy; patients in Group 6 received antimalarial therapy; patient in Group 7 did not receive any treatment; and patients in Group 8 received antibiotics alone.

### 2.5. Statistical analysis

The data were then cleaned and recorded. Descriptive statistics were performed for continuous variables and expressed as mean and standard deviation (SD). The frequency distribution was performed for categorical variables and expressed as numbers and percentages. Mean differences in the length of hospital stay between different treatment groups were analyzed using the Kruskal-Wallis test. The associations between sociodemographic factors, signs, and symptoms between different groups of symptomatic patients were analyzed and are presented below. The Mann–Whitney U test was used to analyze the length of hospital stay compared to treatment groups that could be adjusted for baseline factors (comorbidities and severity of the disease) to determine whether the treatments affected the length of stay. Multiple linear regression was used to compare baseline characteristics between the groups according to the treatment received. Data were analyzed using IBM SPSS version 21 (SPSS version 21.0; IBM Corporation, Armonk, NY, USA). Statistical significance was set at *P* < .05.

## 3. Results

A total of 843 SARS-COV-2-positive patients admitted to the hospital between March 12, 2020, and June 30, 2020, were included in the study. The mean age was 34.95 ± 15.9 years, and 67% of the patients were male. Of the 843 patients, 132 were mildly symptomatic, 168 were moderately symptomatic, and 17 were severely symptomatic. Among the 843 patients, 108 had diabetes mellitus, hypertension was observed in 84 patients, renal failure and coronary artery disease were present in 8 cases separately, heart failure was observed in 7 patients, atrial fibrillation in 2, and chronic obstructive pulmonary disease in 1 patient. Table 1 summarizes the baseline characteristics of the enrolled patients.

**Table 1:** Baseline characteristic distribution of the patients enrolled.

Characteristics	All patients (n* = 843*)	Mild symptomatic cases (n* = 132*)	Moderate symptomatic cases (n* = 168*)	Severe symptomatic cases (n* = 17*)	*P*-value
**Age in years** (mean and SD)	34.95 (15.9)	36.52 (16.06)	36.68 (15.42)	36.94 (15.11)	.128
**Sex**					
Female	278 (33)	35 (26.5)	53 (31.5)	5 (29.4)	.274
Male	565 (67)	97 (73.5)	115 (68.5)	12 (70.6)	
**Comorbidities No. (%**)					
Diabetes mellitus	108 (12.8)	25 (18.9)	26 (15.5)	3 (17.6)	**.031** *
Hypertension	84 (10)	16 (12.1)	18(10.7)	2 (11.8)	.738
Renal failure	8 (0.9)	1 (0.8)	2 (1.2)	–	.957
AF	2 (0.2)	–	–	–	.751
Coronary artery disease	8 (0.9)	–	1 (0.6)	–	.270
HF	7 (0.8)	–	1 (0.6)	–	.578
COPD	1(0.1)	–	–	–	.896
Travel and contact history					
Recent travel	21 (2.5)	4 (3)	7 (4.2)	1 (5.9)	.239
Contact with COVID-19-positive patient	560 (66.4)	87 (65.9)	108 (64.3)	9 (52.9)	.549
Length of hospital stay (mean and SD)	7.71 (5.65)	8.18 (5.62)	7.81 (5.89)	6.82 (4.55)	.662
**Symptoms No. (%**)					
Fever	199 (23.6)	33 (25)	47 (28)	10 (58.8)	**.001** *
Cough	154 (18.3)	28 (21.2)	42 (25)	7 (41.2)	**.001** *
Shortness of breath	131 (15.5)	24 (18.2)	33 (19.6)	5 (29.4)	.053
Sore throat	88 (10.4)	16 (12.1)	16 (9.5)	5 (29.4)	.060
Runny nose	6 (0.7)	1 (0.8)	2 (1.2)	–	.861
Headache	33 (3.9)	7 (5.3)	8 (4.8)	1 (5.9)	.531
Myalgia/fatigue	23 (2.8)	4 (3.1)	8 (4.8)	1 (5.9)	.740
Vomiting	18 (2.1)	3 (2.3)	2 (1.2)	1 (5.9)	.624

* *P*-value considered statistically significant.

AF = atrial fibrillation, COPD = chronic obstructive pulmonary disease, COVID-19 = Coronavirus disease, HF = heart failure.

When comorbidities and other symptoms were compared between groups, most variables were similar in all treatment groups (*P* < .005), except for renal failure for comorbidities and running nose for symptoms (Table 2).

**Table 2:** Comparing baseline characteristics between groups based on treatment received.

Characteristics	Treatment group								*P*-value
	1 (n = 40)	2 (n = 11)	3 (n = 29)	4 (n = 160)	5 (n = 48)	6 (n = 14)	7 (n = 409)	8 (n = 132)	
Age in years (mean and SD)	45.9 (12.5)	50.18 (23)	37.3 (14.3)	37.9 (14.1)	33.1 (16.7)	38.8 (18.1)	31.2 (14.8)	38 (17.6)	.001*
Sex									
Female	13 (32.5)	7 (63.6)	9 (31)	41 (25.6)	20 (41.7)	4 (28.6)	126 (30.8)	58 (43.9)	.010*
Male	27 (67.5)	4 (36.4)	20(69)	119 (74.4)	28 (58.3)	10 (71.4)	283 (69.2)	74 (56.1)	
**Comorbidities No. (%**)									
Diabetes mellitus	10 (25)	4 (36.4)	9 (31)	29 (18.1)	3 (6.3)	4 (28.6)	28 (6.8)	21 (15.9)	.001*
Hypertension	7 (17.5)	3 (27.3)	6 (20.7)	19 (11.9)	2 (4.2)	2 (14.3)	28 (6.8)	17 (12.9)	.011*
Renal failure	−	−	−	2 (1.3)	−	−	−	6 (4.5)	.001*
AF	−	−	−	−	−	−	1 (0.2)	1 (0.8)	.946
Coronary artery disease	−	−	−	2 (1.3)	−	−	−	6 (4.5)	.005*
HF	−	−	−	1 (0.6)	−	−	1 (0.2)	5 (3.8)	.018*
COPD	−	−	1 (3.4)	−	−	−	−	−	.001*
**Travel and contact history**									
Recent travel	2 (5)	−	2 (6.9)	7 (4.4)	2 (4.2)	1 (7.1)	4 (1)	3 (2.3)	.113
Contact with COVID-19-positive patient	23 (57.5)	4 (36.4)	22 (75.9)	121 (75.6)	27 (56.3)	9 (64.3)	254 (62.1)	100 (75.8)	.001*
Length of hospital stay (mean and SD)	16.38 (7.61)	13.18 (7.02)	13.72 (8.28)	9.3 (5.61)	6.96 (3.32)	10.86 (8.05)	5.77 (3.94)	7.37 (4.78)	.001*
**Symptoms No.(%**)									
Fever	29 (72.5)	1 (9.1)	10 (34.5)	83 (51.9)	3 (6.3)	3 (21.4)	27 (6.6)	43 (32.6)	.001*
Cough	25 (62.5)	1 (9.1)	12 (41.4)	54 (33.8)	2 (4.2)	4 (28.6)	21 (5.1)	35 (26.5)	.001*
Shortness of breath	17 (42.5)	1 (9.1)	6 (20.7)	62 (38.8)	2 (4.2)	1 (7.1)	13 (3.2)	29 (22)	.001*
Sore throat	11 (27.5)	1 (9.1)	2 (6.9)	33 (20.6)	6 (12.5)	2 (14.3)	12 (2.9)	21 (15.9)	.001*
Running nose	1 (2.5)	−	−	−	−	1 (7.1)	1 (0.2)	−	.214
Headache	4 (10)	−	2 (6.8)	12 (7.5)	−	1 (7.1)	6 (1.5)	9 (6.9)	.002*
Myalgia/fatigue	3 (7.5)	−	−	4 (2.5)	−	−	3 (0.7)	8 (6.1)	.001*
Vomiting	1 (2.5)	−	2 (6.8)	5 (3.1)	−	−	1 (0.2)	7 (5.3)	.004*

AF = atrial fibrillation, COPD = chronic obstructive pulmonary disease, COVID-19 = Coronavirus disease, HF = heart failure.

When comorbidities and other symptoms were compared between treatment groups, most variables were similar at all different levels of symptomatic cases (*P* > .005), except for diabetes mellitus, fever, and coughing (*P* < .005). The Mann-Whitney test showed that there were significant differences in the length of hospital stay between different treatment groups with chi-square (7) = 156.327, with a *P*-value of .000 (*P*-value < .005). Treatment Group 7, which comprised 409 patients without treatment (48.5%), had fewer hospital stays, with a mean duration of 5.7 days. Treatment Group 7 was considerably better than the other groups and showed a statistically significant difference (Table 3).

**Table 3:** Comparison between treatment groups and hospital stays.

Treatment group	Comparison group	*Z* score	*P*-value
1	2	−1.124	.261
	3	−1.515	.130
	4	−5.408	.000*
	5	−6.100	.000*
	6	−2.441	.015*
	7	−8.621	.000*
	8	−6.704	.000*
2	3	−.137	.891
	4	−1.809	.070
	5	−2.709	.007*
	6	−.906	.365
	7	−3.689	.000*
	8	−2.851	.004*
3	4	−2.782	.005*
	5	−3.867	.000*
	6	−1.171	.242
	7	−5.807	.000*
	8	−4.155	.000*
4	5	−2.404	.016*
	6	−.494	.621
	7	−7.771	.000*
	8	−3.226	.001*
5	6	−1.357	.175
	7	−3.013	.003*
	8	−.268	.789
6	7	−2.734	.005*
	8	−1.527	.127
7	8	−3.657	.000*

**Figure 1.**
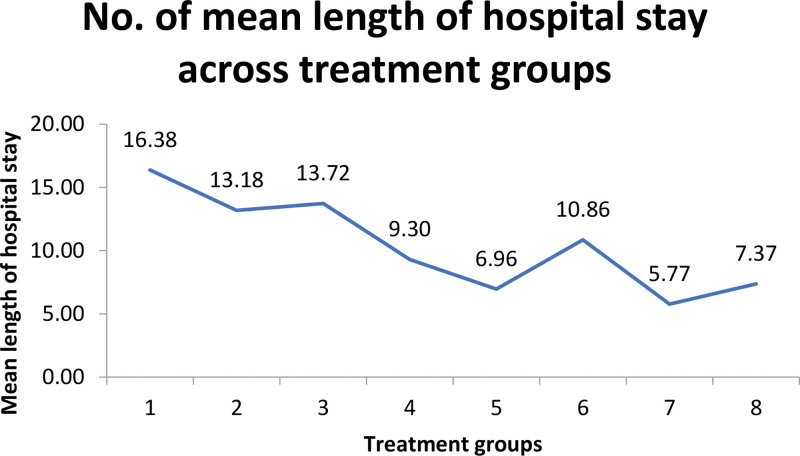
Mean length of hospital stay across treatment groups.

The mean length of hospital stay was calculated for all treatment groups. The mean duration of hospital stays for Groups 1 to 8 was 16.38, 13.18, 13.72, 9.30, 6.96, 10.86, 5.77, and 7.37 days, with 156.327 chi-squares and .000* *P*-value (see Table 3). Group 1 had the highest mean length of hospital stay, while Group 7 had the shortest stay (Table 3). Figure 1 shows a graphical representation of the mean length of hospital stay between treatment groups.

The Z score and *P*-value were calculated for all treatment and comparison groups to describe the relationships between the groups. Group 1 showed a significant difference in the length of hospital stay compared to Groups 4, 5, 6, 7, and 8. Group 2 showed a significant difference in the length of hospital stay compared to Groups 5, 7, and 8. Group 3 showed a significant difference in the length of hospital stay compared to Groups 4, 5, 7, and 8. Group 4 showed a significant difference in hospital stay compared to Groups 5, 7, and 8. Group 5 showed a significant difference in hospital stay compared to Group 7. Group 6 showed a significant difference in hospital stay compared to Group 7. Group 7 showed a significant difference in hospital stay compared to Group 8 (See Table 4).

**Table 4:** Hospital length of stay between the treatment groups adjusted for (comorbidities and severity of the disease).

Model	Unstandardized coefficients		Standardized Coefficients	t	Sig.	95% confidence interval for B	
	B	Std. Error	Beta			Lower Bound	Upper Bound
(Constant)	−3.886	10.086		−0.385	0.701	−23.788	16.016
Diabetes Mellitus	−1.090	1.183	−0.065	−0.921	0.358	−3.423	1.244
Hypertension	−1.159	1.349	−0.058	−0.859	0.391	−3.820	1.503
RF	5.496	3.882	0.145	1.416	0.159	−2.164	13.155
AF	.480	4.192	0.007	.115	0.909	−7.792	8.753
CAD	−9.400	4.676	−0.264	−2.010	0.046*	−18.627	−0.173
HF	11.166	5.087	0.273	2.195	0.029*	1.129	21.204
Treatment 1	11.646	1.474	0.511	7.901	0.000*	8.738	14.555
Treatment 2	10.898	5.858	0.110	1.860	0.064	−0.661	22.457
Treatment 3	9.841	1.809	0.347	5.440	0.000*	6.271	13.411
Treatment 4	4.487	1.112	0.272	4.036	0.000*	2.293	6.681
Treatment 5	4.293	3.017	0.086	1.423	0.157	−1.661	10.247
Treatment 6	2.999	2.539	0.073	1.181	0.239	−2.011	8.009
Treatment 8	1.508	1.301	0.082	1.159	0.248	−1.058	4.075
Mild symptoms patient	−2.205	1.245	−0.114	−1.771	0.078	−4.662	0.251
Moderate symptoms patient	−1.171	1.066	−0.072	−1.098	0.273	−3.274	0.933
Severe symptoms patient	−3.476	1.970	−0.108	−1.764	0.079	−7.363	0.412

AF = atrial fibrillation; HF = heart failure.

Multiple linear regression was used to test whether different treatment groups and comorbidities significantly predicted the length of hospital stay. Patients with heart failure tended to have a longer hospital stay (*P* = .029 as well as patients in treatment Groups 1, 3, and 4 (*P* < .005). The overall regression model was statistically significant [*R*^2^ = 0.379; F(16,180) = 6.875; *P* = .001*) (Table 5).

**Table 5:** Hospital length of stay between the treatment groups adjusted for (comorbidities and severity of the disease).

Model	Unstandardized coefficients		Standardized Coefficients	t	Sig.	95% confidence interval for B	
	B	Std. Error	Beta			Lower Bound	Upper Bound
(Constant)	−3.886	10.086		−0.385	0.701	−23.788	16.016
Diabetes Mellitus	−1.090	1.183	−0.065	−0.921	0.358	−3.423	1.244
Hypertension	−1.159	1.349	−0.058	−0.859	0.391	−3.820	1.503
RF	5.496	3.882	0.145	1.416	0.159	−2.164	13.155
AF	.480	4.192	0.007	.115	0.909	−7.792	8.753
CAD	−9.400	4.676	−0.264	−2.010	0.046*	−18.627	−0.173
HF	11.166	5.087	0.273	2.195	0.029*	1.129	21.204
Treatment 1	11.646	1.474	0.511	7.901	0.000*	8.738	14.555
Treatment 2	10.898	5.858	0.110	1.860	0.064	−0.661	22.457
Treatment 3	9.841	1.809	0.347	5.440	0.000*	6.271	13.411
Treatment 4	4.487	1.112	0.272	4.036	0.000*	2.293	6.681
Treatment 5	4.293	3.017	0.086	1.423	0.157	−1.661	10.247
Treatment 6	2.999	2.539	0.073	1.181	0.239	−2.011	8.009
Treatment 8	1.508	1.301	0.082	1.159	0.248	−1.058	4.075
Mild symptoms patient	−2.205	1.245	−0.114	−1.771	0.078	−4.662	0.251
Moderate symptoms patient	−1.171	1.066	−0.072	−1.098	0.273	−3.274	0.933
Severe symptoms patient	−3.476	1.970	−0.108	−1.764	0.079	−7.363	0.412

AF = atrial fibrillation, HF = heart failure.

## 4. Discussion

COVID-19 has threatened the world since December 2019 and has been declared a global pandemic in all healthcare facilities worldwide. The spectrum of diseases caused by COVID-19 ranges from mild to critical. This study included data from 2 medical centers in Taif City, KSA, to summarize the length of hospital stay as well as the clinical and therapeutic outcomes of patients positive for COVID-19. The length of hospital stay and clinical and therapeutic characteristics of patients with COVID-19 are not only crucial indicators of the availability of healthcare services, but also provide the necessary information on quality care for healthcare management and future requirements. Lower hospital stay is associated with better progress in treatment, higher quality of care, lower healthcare costs, and a lower risk of hospital-acquired infections.^[22]^

This is brought back to our attention because a new subvariant of SARS-CoV-2, XBB, was dramatically announced earlier this month in Singapore. The same subvariant has also appeared in Hong Kong. A highly mutated descendant of the Omicron variant of SARS-CoV-2 that led to a recorded wave of infections starting approximately a year ago, XBB is, in many ways, the worst form of the virus so far. It is more contagious than any previous variant or subvariant. It also escapes antibodies from monoclonal therapies, potentially rendering the whole category of drugs ineffective as COVID treatments. Although XBB appears to be gaining traction in Asia, a close cousin of XBB, called BQ.1.1, is spreading rapidly in Europe and some in the USA. There are other disputes, including BA.2.75.2. Immune escape is a common feature. At least 2 of the XBB and BQ.1.1 are much unrecognizable for existing antibody therapies and somewhat less recognizable for the antibodies produced by prime doses of the leading messenger RNA vaccines.^[23,24]^

In our study, male patients were more prevalent than female patients with COVID-19. This varied prevalence might be due to immunological variations in sex or behavioral lifestyle patterns such as smoking.^[25]^ Diabetes mellitus was the most common comorbidity, followed by hypertension. The mean length of stay was 7.7 ± 5.6 days; 842 patients were discharged, 18 were intubated, and 1 died. In this study, the mean number of drugs prescribed (mean ± SD) was 4.7 ± 3.2. Compared to the study with hydroxychloroquine and azithromycin as treatments for COVID-19, the number of antibiotic-treated patients was 418 (49.6).^[26]^ Of the patients, 6 were asymptomatic, 22 had symptoms of upper respiratory tract infection, and 8 had symptoms of lower respiratory tract infection. Twenty patients were treated and showed a significant reduction in viral load on day 6-post inclusion compared to controls and a much lower average carrying duration. The addition of azithromycin to hydroxychloroquine was significantly more efficient for virus elimination. In our study, 258 (30.6%) patients were on antiviral therapy, which is consistent with the outcome of the study. The use of remdesivir to treat patients in hospitals with COVID-19 in Canada^[27]^ that can procure remdesivir, compared to standard of care, has a modest but significant effect on important outcomes for patients and health systems, such as the need for mechanical ventilation.

The efficacy of antimalarial treatment (hydroxychloroquine) in alleviating acute respiratory symptoms was 95 (11.3%). In a study on the safety and efficacy of hydroxychloroquine for the treatment of non-severe COVID-19,^[28]^ the results showed that 400 mg of hydroxychloroquine was administered twice daily for the first day, followed by 200 mg twice daily for the next 4 days, and was safe but not associated with a reduction in viral load or symptoms improvement among adults with COVID-19 in Uganda. In our study, 45 (5.3%) patients received corticosteroids, as shown in a study on the clinical characteristics and outcomes of critically ill mechanically ventilated patients with COVID-19 who received interleukin-6 receptor antagonist (IL-6RA) and corticosteroid therapy.^[29]^ On day 28, compared with steroid use alone, IL-6RA use was associated with an adjusted incidence rate ratio (aIRR) of 1.12 (95% confidence interval [CI], 0.88, 1.4) for ventilator-free days, while combination therapy was associated with an aIRR of 0.83 (95% CI, 0.6–1.14). The use of IL-6RA was associated with an adjusted odds ratio (aOR) of 0.68 (95% CI, 0.44–1.07) for the 28-day mortality rate, while a combination therapy of IL-6RA and steroids was associated with an aOR of 1.07 (95% CI, 0.67–0.70).^[28]^

The treatment group with antiviral, antimalarial, and antibiotic therapy had the highest mean length of hospital stay (16.38 days). This was followed by patients in Group 2, who received antiviral and antimalarial therapy and Group 3, who received antimalarial and antibiotic therapy, at 13.18 and 13.72 days of hospital admission, respectively.

The group that received no treatment (Group 7) had the shortest hospital stay, 5.77 days. Patients in Group 5, who received antiviral therapy had 6.96 days of hospital stay; patients in Group 8, who received antibiotics alone, had 7.37 days; patients in Group 4, who received antiviral and antibiotic therapy, had 9.30 days; and patients in Group 6, who received antimalarial therapy, had 10.86 days of hospital admission. One crucial finding of our study suggested that antimalarial therapy, either alone or in combination, had an increase in the length of hospital stay. In fact, the use of antimalarial in combination therapy prolongs the length of hospital stay.

Data from this study were collected at the beginning of the COVID-19 pandemic, and they have some limitations. The study was conducted when the vaccine had not yet circulated in the population. Almost 2-thirds of the adult population currently receive the vaccine in the KSA, and the result could be fitted to the non-vaccinated population. In the early pandemic, there was no defined treatment for COVID-19, and supportive treatment and ventilation support were available according to the severity of the disease and symptoms.^[25]^ Although there is currently an FDA-approved new antiviral for COVID-19 (remdesivir, paxlovid, and molnupiravir), it could be coordinated in some cases.

This study provides insights based on data from patients with COVID-19 in 2 healthcare centers in Taif City, KSA; low mortality is a quality indicator of the early response to a pandemic. However, a comprehensive management approach is required.

## 5. Conclusions

The length of hospital stay and clinical and therapeutic characteristics of patients with COVID-19 are crucial indicators of pandemic management. A shorter hospital stay is a positive outcome for better management of COVID-19.

## Acknowledgments

We would like to thank Editage (www.editage.com) for English language editing.
